# Potential causes of the preoperative increase in the rectosigmoid cyclic motor pattern: A high‐resolution manometry study

**DOI:** 10.14814/phy2.15091

**Published:** 2021-11-27

**Authors:** Cameron I. Wells, Sameer Bhat, Nira Paskaranandavadivel, Anthony Y. Lin, Ryash Vather, Chris Varghese, James A. Penfold, David Rowbotham, Phil G. Dinning, Ian P. Bissett, Greg O’Grady

**Affiliations:** ^1^ Department of Surgery The University of Auckland Auckland New Zealand; ^2^ Auckland Bioengineering Institute The University of Auckland Auckland New Zealand; ^3^ Department of General Surgery Capital and Coast District Health Board Wellington New Zealand; ^4^ Department of Colorectal Surgery Royal Adelaide Hospital Adelaide South Australia Australia; ^5^ Department of Gastroenterology and Hepatology Auckland District Health Board Auckland New Zealand; ^6^ Discipline of Human Physiology Flinders University Adelaide South Australia Australia; ^7^ Department of Gastroenterology Flinders Medical Centre Bedford Park South Australia Australia; ^8^ Department of Surgery Auckland District Health Board Auckland New Zealand

**Keywords:** colonic manometry, motility, oral carbohydrate, preoperative

## Abstract

**Background:**

Cyclic motor patterns (CMPs) are the most common motor pattern in the distal colon. This study used high‐resolution (HR) colonic manometry to quantify trends in distal colonic motor activity before elective colonic surgery, determine the effect of a preoperative carbohydrate load, and compare this with a meal response in healthy controls.

**Methods:**

Fiber‐optic HR colonic manometry (36 sensors, 1 cm intervals) was used to investigate distal colonic motor activity in 10 adult patients prior to elective colonic surgery, 6 of whom consumed a preoperative carbohydrate drink (200 kCal). Data were compared with nine healthy volunteers who underwent HR colonic manometry recordings while fasted and following a 700 kCal meal. The primary outcome was the percentage of recording occupied by CMPs, defined as propagating contractions at 2–4 cycles per minute (cpm). Secondary outcomes included amplitude, speed, and distance of propagating motor patterns.

**Results:**

The occurrence of CMPs progressively increased in time periods closer to surgery (*p* = 0.001). Consumption of a preoperative drink resulted in significantly increased CMP occurrence (*p* = 0.04) and propagating distance (*p* = 0.04). There were no changes in amplitude or speed of propagating motor patterns during the preoperative period. The increase in activity following a preoperative drink was of similar magnitude to the colonic meal response observed in healthy controls, despite the lesser caloric nutrient load.

**Conclusion:**

Distal colonic CMP increased in occurrence prior to surgery, amplified by ingestion of preoperative carbohydrate drinks. We hypothesize that anxiety, which is also known to rise with proximity to surgery, could play a contributing role.

## Graphical abstract

Distal colonic cyclic motor patterns increased in occurrence prior to surgery, amplified by ingestion of preoperative carbohydrate drinks. We hypothesize that anxiety, which is also known to rise with proximity to surgery, could play a contributing role.

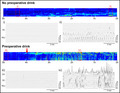

## 1 BACKGROUND

Distal colonic cyclic motor patterns (CMPs) are the dominant motor pattern identified in the human colon by high‐resolution (HR) colonic manometry (Dinning et al., 2014; Lin, Du, et al., 2017; Pervez et al., 2020). The CMP is characterized by propagating contractions at 2–6 cycles per minute (cpm) occurring in clusters in the distal colon, often in a retrograde direction, and is hypothesized to act as a rectosigmoid brake, helping to control rectal filling and preserve continence (Lin et al., 2017b; Lin, Du, et al., 2017). Recent advances in the understanding of distal colonic motor activity have been achieved using HR colonic manometry, which has provided spatiotemporal detail that was previously unattainable (Dinning et al., 2013, 2014). These studies have demonstrated the importance of the CMP in both healthy physiology and pathological states (Keane et al., 2021; Lin, Du, et al., 2017; Pervez et al., 2020; Vather et al., 2018).

Despite the importance of CMPs in colonic function, their regulation remains incompletely understood. In healthy controls, CMP activity increases following a meal as part of the gastrocolic reflex (Dinning et al., 2014; Huizinga et al., 2021; Lin, Du, et al., 2017; Pervez et al., 2020). However, a previous study by our group in patients undergoing colorectal surgery also showed an interesting and substantial progressive increase in CMPs during fasted preoperative recordings, potentially attributable to progressively increasing anxiety levels before surgery (Badner et al., 1990; Jawaid et al., 2007; Vather et al., 2018). While that study was conducted in fasted patients, more recently recruited patients have received a preoperative carbohydrate drink as part of an Enhanced Recovery After Surgery (ERAS) protocol to reduce perioperative insulin resistance and subsequent complications (Amer et al., 2017; Gustafsson et al., 2019). This change in perioperative practice therefore provides an ideal context to evaluate the effects of a nutrient load on distal colonic motility in the context of the well‐known anxiety‐provoking stimulus of impending surgery (Badner et al., 1990; Jawaid et al., 2007).

Therefore, this study aimed to use HR colonic manometry to: (i) further confirm and quantify trends in distal colonic motor activity occurring in the preoperative period before elective major colorectal surgery, (ii) determine the effect of a preoperative carbohydrate drink compared with fasting on distal colonic motor activity in this context, and (iii) compare this to a meal response obtained from healthy controls.

## 2 METHODS

Ethical approval was obtained from the New Zealand Health and Disability Ethics Committee and the Flinders University Human Research Ethics Committee. All participants provided informed written consent.

### 2.1 Eligibility criteria

Adult patients aged >18 years old who were planned for elective right‐sided colonic resection at Auckland City Hospital (Auckland District Health Board, Auckland, New Zealand) were eligible for inclusion. Recruitment occurred during two time periods: August 2013 to September 2014, and May 2017 to September 2019. Exclusion criteria included previous left‐sided colorectal resection, documented history of current or previous functional gastrointestinal disorder (FGID), or comorbidities known to affect GI motility. All patients had a normal daily bowel habit at baseline; none had prior symptoms consistent with IBS or other functional motility disorders.

### 2.2 High‐resolution colonic manometry

A fiber‐optic HR manometry catheter with 36 sensors at 1 cm intervals was used to measure distal colonic motor activity (Arkwright et al., 2009). During the recordings, the catheter was connected to a spectral interrogator acquisition unit (FBG‐scan 804; FOS&S, Geel, Belgium). A purpose‐written LabVIEW program (National Instruments) was used to record data.

### 2.3 Perioperative protocol

Participants presented to the endoscopy suite between 7 and 8 am on the morning of their planned operation, and were fasted since the previous midnight. Two fleet enemas were administered prior to catheter insertion; no patient underwent oral mechanical bowel preparation. The fiber‐optic HR manometry catheter was endoscopically inserted and secured in the distal colon as previously described (Vather et al., 2018). All procedures were performed under either light sedation with midazolam and fentanyl, or no sedation.

In the first study period between 2013 and 2014, patients were kept nil by mouth preoperatively, and received no food or drink during the preoperative recording period. In the second study period between 2017 and 2019, the institutional ERAS protocol had been updated to include the routine use of preoperative carbohydrate drinks. During these studies, patients were given two preoperative carbohydrate drinks (Nutricia preOp®; Numico) at approximately 2 h (h) prior to surgery (total volume of 400 ml and caloric content of 200 kCal). Three patients (two of whom received a preoperative carbohydrate drink) received premedication with 1 g of oral paracetamol during the recordings. Patients did not receive any other medications or interventions during the preoperative recordings, and there were no other changes in the preoperative care received by patients during the two study periods. Other than manometry recordings, standard preoperative care was received by all patients.

Preoperative manometric recordings were obtained once patients were awake and recovered from any sedation used during insertion for a period of at least 30 min. Patients were awake for the duration of the preoperative recordings, which continued while the patient was in the endoscopy unit recovery area, and in the preoperative assessment unit. Patients underwent at least 3 h of preoperative recordings before proceeding for their planned operation. Continuous recordings were obtained, except when disconnection was necessitated for patient transfers. Manometry recordings were also obtained intra‐ and post‐operatively, however these data have been reported in detail elsewhere (Vather et al., 2018), and were not considered as part of this analysis. Recordings obtained in the operating room prior to the beginning of surgery were not included in this analysis due to the potential confounding effects of anesthetic agents on colonic motor activity (Condon et al., 1987).

### 2.4 Healthy controls

Control data from healthy volunteers were included to provide an example of physiological colonic activity with a standardized meal response (Dinning et al., 2014; Lin, Du, et al., 2017). These participants received oral mechanical bowel preparation, and were given a 700 kCal meal, consisting of 300 ml of TwoCal® HN Vanilla (Abbott Nutrition) and a chicken sandwich, with 2 h of preprandial and 2 h of postprandial HR colonic manometry recordings. Recordings in these healthy controls started between 09:00 am and 11:30 am. A fiber‐optic manometry catheter with 72 sensors at 1 cm intervals was used in these patients, though for consistency in comparisons with the preoperative cohort during analysis, only motor events from the most distal 36 sensors (i.e., those located in the descending colon, sigmoid colon, and rectum) were evaluated. High‐amplitude propagating contractions (HAPCs) were only observed in the healthy control data and were therefore not further compared in this study.

### 2.5 Colonic manometry analysis

Manometry data were analyzed using a previously described automated framework developed specifically for fiber‐optic HR manometry (Paskaranandavadivel et al., 2021). Channels occupied by the anal sphincter were manually identified and removed. Baseline drift was removed using an envelope detection technique, followed by the suppression of synchronous events and high‐frequency noise (Paskaranandavadivel et al., 2021). Propagating contractions were detected using a threshold of 10 mmHg to identify individual pressure peaks, which were then grouped into the propagating wave front, consisting of propagation of 4 cm or more (Paskaranandavadivel et al., 2021). Quantitative metrics denoting the amplitude, speed, and direction of travel (antegrade vs. retrograde) were then computed. Frequency of the pressure events was also calculated as the reciprocal of the time period across pressure events in the propagating contraction. Amplitude was defined as the average maximal pressure deflection in all channels occupied by a propagating pressure wave (Paskaranandavadivel et al., 2021; Vather et al., 2018).

For the purposes of this analysis, preoperative recordings were divided into 0–1 h, 1–2 h, 2–3 h, and 3–4 h periods prior to surgery. In patients who received a preoperative carbohydrate drink, recordings were also segmented into pre‐ and post‐drink periods. Similarly, for healthy control subjects, recordings were divided into pre‐ and post‐prandial periods.

### 2.6 Outcome measures

The primary outcome was percentage of recording duration occupied by CMP, defined as propagating colonic motor activity occurring in the 2–4 cpm range. Secondary outcomes included the direction (antegrade vs. retrograde), amplitude, distance, and speed of propagating motor patterns.

### 2.7 Statistical analysis

Analysis was conducted using R Studio (Version 1.4.1106). Descriptive statistics were used to summarize and display data. Continuous data were presented as mean ± standard deviation (SD), unless otherwise stated, and categorical data were presented as frequency (n) and proportion (%). Skewed continuous variables were log‐transformed, and continuity corrections of +1 were applied where appropriate. A mixed two‐way analysis of variance (ANOVA) was performed to evaluate the effects of preoperative timing (within‐subjects) and preoperative drink (between‐subjects) on the outcomes of interest from HR colonic manometry. A linear mixed‐effects model was fitted using the restricted maximum likelihood method with each participant as the random effect. P‐values were calculated via the Satterthwaite's degrees of freedom method (Kuznetsova et al., 2017). Results of the linear mixed model are presented as exponentiated beta coefficients with their corresponding 95% confidence intervals (CIs). The paired samples Student's *t*‐test was used to compare changes following a meal or preoperative carbohydrate drink in healthy control subjects and surgical patients, respectively. An independent samples Student's *t*‐test was used to compare midazolam and fentanyl doses received by patients who did and did not receive a preoperative carbohydrate drink. P‐values less than 0.05 were considered statistically significant.

## 3 RESULTS

### 3.1 Participant selection and characteristics

Ten patients undergoing elective right‐sided colectomy were included, together with nine healthy controls. The surgical cohort was older and had more males compared with the healthy controls (Table 1). Preoperative carbohydrate drinks were consumed by seven patients, at a mean of 3.2 ± 1.4 h prior to surgery. Patients with and without a preoperative drink received similar doses of fentanyl (mean 58.3 ± 64.5 vs. 25.0 ± 50.0 mcg, *p* = 0.39) and midazolam (mean 1.2 ± 1.0 vs. 0.5 ± 1.0 mg, *p* = 0.34) during the endoscopic insertion of the HR manometry catheter.

**TABLE 1:** Demographic and clinical factors of included participants

	CHO drink (*n* = 6)	No CHO drink (*n* = 4)	Healthy controls (*n* = 9)
Age [years], median (range)	74 (26–84)	66 (52–73)	51 (30–65)
Male gender, n (%)	55 (83%)	3 (75%)	3 (33%)
Pathology	Neoplasia =5 and Stricture =1	Neoplasia =4	—

Abbreviations: CHO, carbohydrate; n, number.

### 3.2 High‐resolution colonic manometry

Patients had a mean 4.5 ± 1.6 h of HR colonic manometry recordings preoperatively (4.5 ± 1.2 h in the drink cohort vs. 5.0 ± 1.8 h in the no‐drink cohort). Healthy control subjects had a mean fasted and postprandial manometry recording duration of 2.4 ± 0.2 h and 2.4 ± 0.5 h, respectively, compared to a mean 1.4 ± 0.8 h prior to and 3.2 ± 1.4 h following the preoperative carbohydrate drink in surgical patients.

### 3.3 Preoperative data

**FIGURE 1:**
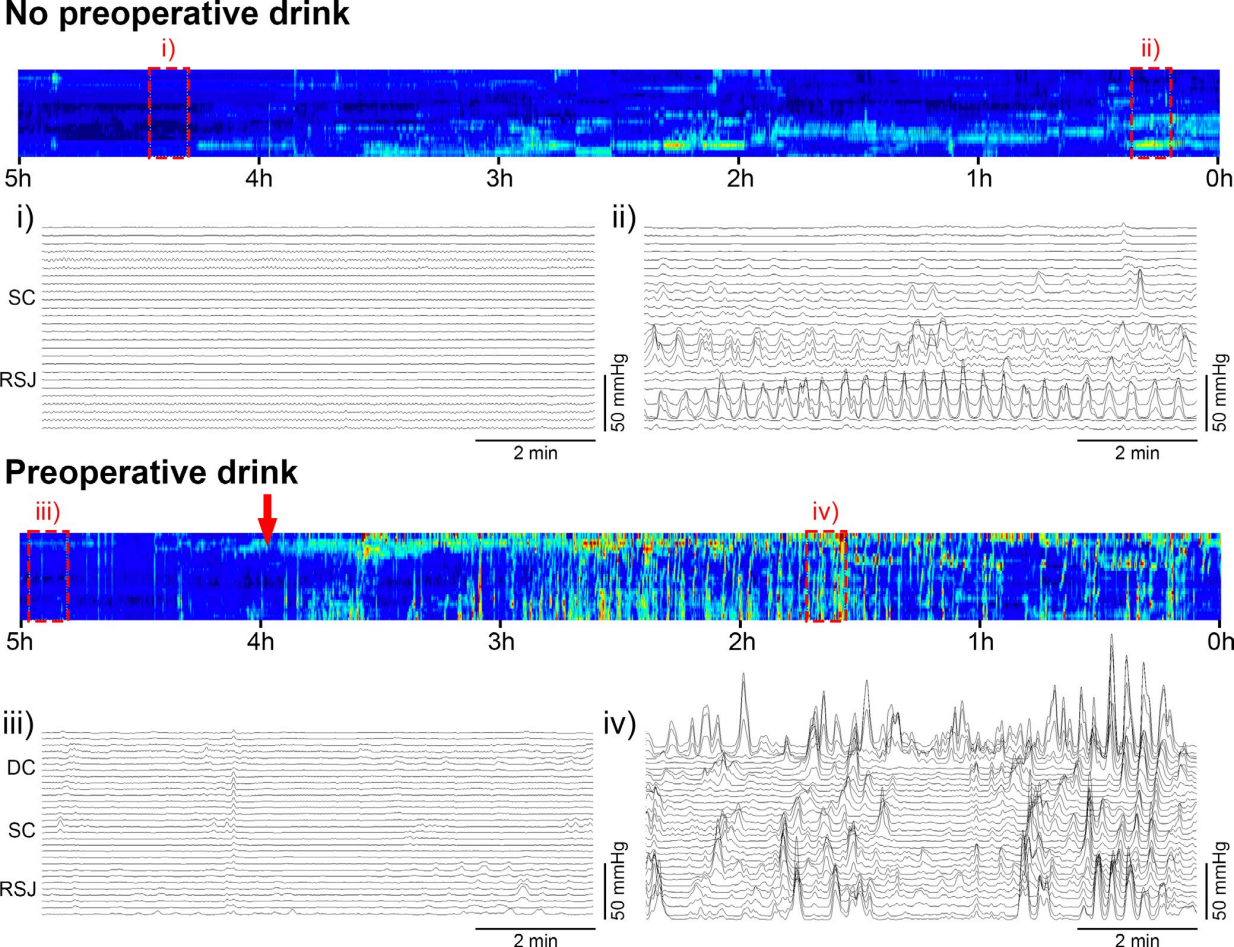
Examples of preoperative cyclic motor patterns occurring in patients undergoing elective right‐sided colonic resection without (top) and with (bottom) a 200 kCal preoperative carbohydrate drink. DC, descending colon; RSJ, rectosigmoid junction; SC, sigmoid colon

**FIGURE 2:**
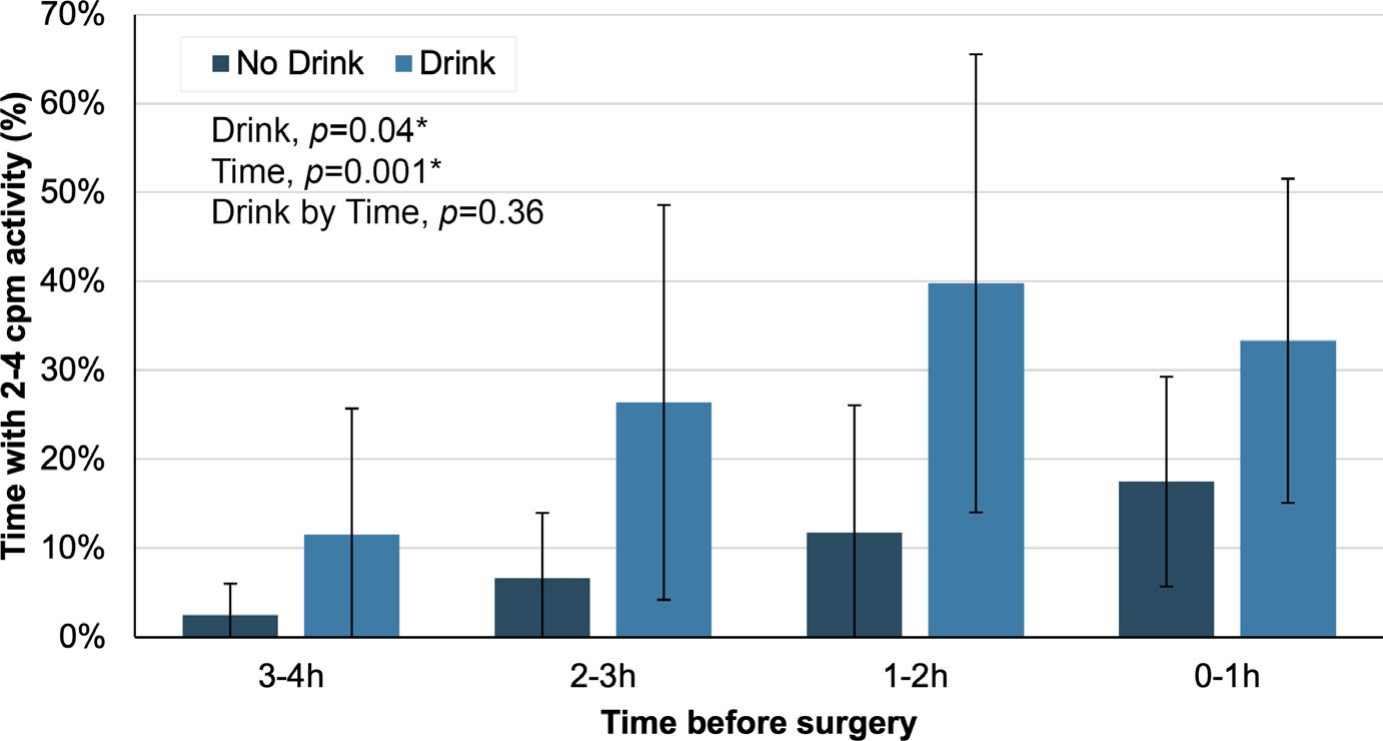
Percentage of recording duration with 2–4 cpm motor activity during the preoperative recordings, stratified according to use of a 200 kCal preoperative carbohydrate drink, in patients undergoing elective right‐sided colonic resection. The effects of time (*p* = 0.001) and preoperative carbohydrate drink (*p* = 0.04) were assessed using mixed two‐way ANOVA

CMPs were the most common colonic motor events preoperatively, and increased in occurrence during time periods closer to surgery (Figure 1). Propagating contractions occurred at dominant frequencies of 2–4 cpm; higher frequency activity at 8–12 cpm was not observed. At 3–4 h before surgery, 2–4 cpm activity was present for 11.6 ± 14.2% and 2.5 ± 3.5% of the recording in the drink and no‐drink cohorts, respectively. Mixed two‐way ANOVA demonstrated that the occurrence of 2–4 cpm activity progressively increased during the time periods closer to surgery (*p* = 0.001), reaching 17.5 ± 11.8% in the no‐drink cohort and 33.3 ± 18.2% in the drink cohort during the hour immediately prior to surgery (Figure 2). Consumption of a preoperative carbohydrate drink also significantly increased the occurrence of 2–4 cpm activity during the preoperative recordings (*p* = 0.04). A linear mixed model corroborated these results, demonstrating independent effects of preoperative timing, and a preoperative CHO drink on 2–4 cpm activity (Table 2).

**TABLE 2:** Linear mixed model investigating the effects of preoperative CHO drink and timing prior to surgery on the occurrence of 2–4 cpm distal colonic motor patterns

Variable	Exp(Beta)	95% CI	*p*
Drink	3.45	1.49–7.97	0.01^*^
Timing (vs. 3–4 h)			
2–3 h	1.29	0.76–2.17	0.30
1–2 h	1.91	1.13–3.22	0.02^*^
0–1 h	2.52	1.50–4.25	0.001^*^

* Statistically significant *p*‐value (<0.05).

The mean number of total, antegrade, and retrograde motor patterns occurring per hour also increased in time periods closer to surgery and with consumption of a preoperative drink (Figure S1A and S2).

Consumption of a preoperative carbohydrate drink was associated with a significant increase in the distance of propagation of motor patterns in the distal colon (*p* = 0.04). However, neither the time period prior to surgery nor the consumption of a preoperative carbohydrate drink demonstrated an association with the amplitude or speed of propagating distal colonic motor patterns. Preoperative trends in the amplitude, distance, and speed of propagating motor patterns in the distal colon in patients with and without a preoperative carbohydrate drink are displayed in Table 3.

**TABLE 3:** Preoperative amplitude, distance, and speed of propagating distal colonic motor patterns over time in patients undergoing elective right‐sided colonic resection, stratified according to the use of a 200 kCal preoperative carbohydrate drink

	Preoperative time before surgery (hours)				*p*‐value
	3–4 h	2–3 h	1–2 h	0–1 h	
Amplitude (mmHg)					
CHO drink	31.3 ± 13.6	29.7 ± 9.9	30.4 ± 6.9	32.1 ± 7.1	Drink = 0.30
No CHO drink	24.7 ± 7.0	28.9 ± 7.5	25.8 ± 0.7	25.0 ± 4.0	Timing = 0.86 Interaction = 0.54
Distance (cm)					
CHO drink	6.3 ± 1.1	6.8 ± 1.5	6.9 ± 1.6	6.8 ± 1.1	Drink = 0.04*
No CHO drink	5.2 ± 0.5	5.5 ± 1.1	5.5 ± 0.8	5.0 ± 0.4	Timing = 0.51 Interaction = 0.73
Speed (cm/s)					
CHO drink	3.1 ± 0.7	3.2 ± 0.8	3.4 ± 0.9	3.6 ± 0.6	Drink = 0.52
No CHO drink	3.6 ± 1.7	3.8 ± 1.4	3.6 ± 1.5	4.1 ± 1.2	Timing = 0.06 Interaction = 0.63

Abbreviations: CHO, carbohydrate; *p*, probability value.

* Statistically significant *p*‐value (<0.05).

### 3.4 Comparison with healthy controls

**FIGURE 3:**
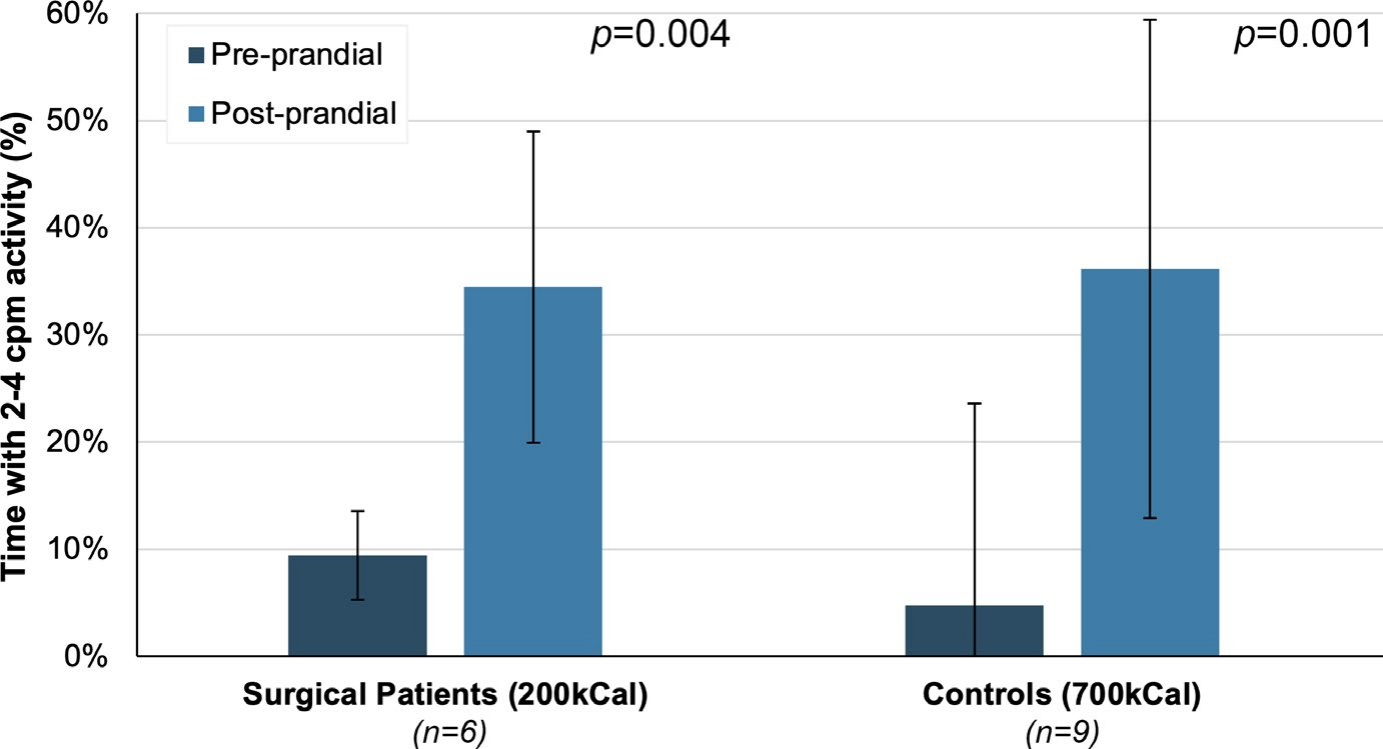
Percentage of recording duration with 2–4 cpm motor activity, before and after a 200 kCal preoperative carbohydrate drink in patients prior to elective right‐sided colonic resection, and fasted and post‐prandially following ingestion of a 700 kCal meal in healthy control subjects. Post‐prandial changes were significant in both the surgical patients (*p* = 0.004) and healthy controls (*p* = 0.001) assessed by the repeated measures Student's *t*‐test

In surgical patients, a significant increase in 2–4 cpm activity was also observed following ingestion of a 200 kCal drink, from 9.4 ± 14.5% pre‐drink to 34.5 ± 23.3% post‐drink (*p* = 0.004) (Figure 3). In comparison, in healthy controls, there was a significant increase in 2–4 cpm motor activity following a 700 kCal meal, from 4.8 ± 4.2% pre‐prandially to 36.2 ± 18.8% post‐prandially (*p* = 0.001). Similar trends in the total number of motor events per hour were observed in surgical patients (*p* = 0.007), and healthy controls (*p* < 0.0001) (Figure S1B), and for both antegrade (surgical patients, *p* = 0.04; controls, *p* < 0.0001) and retrograde (surgical patients, *p* = 0.01; controls, *p* = 0.0002) events, separately (Figure S3).

A significant change in the amplitude, distance, or speed of propagation was not observed following ingestion of a 200 kCal preoperative carbohydrate drink in patients undergoing elective right‐sided colonic resection. In healthy controls, following a 700 kCal meal, both the amplitude (*p* = 0.005) and distance (*p* = 0.0001) of motor events increased post‐prandially, similar to previous manual analysis of this same cohort (Table 4) (Dinning et al., 2014).

**TABLE 4:** Change in the amplitude, distance, and speed of propagating distal colonic motor patterns in healthy subjects and patients undergoing elective right‐sided colonic resection following a 700 kCal meal or 200 kCal preoperative carbohydrate drink.

	Healthy controls			Surgical patients		
	Pre‐meal	Post‐meal	*p*‐value	Pre‐CHO drink	Post‐CHO drink	*p*‐value
Amplitude (mmHg)	21.0 ± 4.2	25.2 ± 3.6	0.005*	26.7 ± 10.5	29.3 ± 5.5	0.49
Distance (cm)	6.9 ± 1.4	9.1 ± 1.6	0.0001*	6.1 ± 1.3	7.0 ± 1.1	0.17
Speed (cm/s)	3.43 ± 1.1	3.1 ± 0.2	0.37	3.3 ± 0.8	3.6 ± 0.9	0.27

Abbreviations: CHO, carbohydrate; *p*, probability value.

* Statistically significant *p*‐values (<0.05).

## 4 DISCUSSION

This study has shown that the occurrence of propagating contractions at 2–4 cpm in the distal colon progressively increased with proximity to surgery. Ingestion of a preoperative carbohydrate drink also independently increased CMP activity. The change in occurrence of cyclic motor patterns following a preoperative carbohydrate drink (200 kCal) in surgical patients was similar in magnitude to that observed before and after a 700 kCal meal in healthy controls. While this may suggest that anxiety prior to surgery may amplify the response to a lower caloric nutrient load, it is also important to note that meal response to a 700 kCal meal starts within seconds of starting the meal, which suggests the colonic response is not entirely calorie dependent. HAPCs were not observed in any preoperative recordings, regardless of whether patients received a preoperative carbohydrate drink. The propagating distance of motor patterns was significantly greater in patients who consumed a preoperative carbohydrate drink, although differences in other motor pattern characteristics were not observed between drink and non‐drink patients.

The factors regulating distal colonic CMP activity remain incompletely understood. HR manometry studies have shown that CMP occurrence increases in both sympathetic (e.g., anxiety and after major surgery) and parasympathetic dominant states (e.g., post‐prandially) (Lin, Dinning, et al., 2017; Vather et al., 2018). CMP can also be experimentally stimulated by proximal colonic distension and the rectal administration of bisacodyl (Pervez et al., 2020). CMPs are thought to arise from a complex interaction between interstitial cells of Cajal (ICC) and the enteric nervous system (ENS), with modulation by autonomic inputs.

As surgery is a high‐stress event, with very high and increasing levels of anxiety known to occur from the afternoon prior to surgery to the immediate preoperative period (Badner et al., 1990; Jawaid et al., 2007), our results lead us to the hypothesis that anxiety may affect preoperative colonic CMP activity. Multiple historical low‐resolution manometry studies have demonstrated that motility patterns in the sigmoid colon may be influenced by stress and anxiety states (Fukudo et al., 1993; Lind, 1991; Welgan et al., 1985, 1988). Almy et al. conducted a series of seminal studies in the 1940’s using balloon kymography, and demonstrated that sigmoid motility was increased by experimentally induced stress in healthy volunteers, but that patients with IBS had variable motility responses (Almy et al., 1949, 1950; Almy & Tulin, 1947). On close inspection, the recordings presented in these papers demonstrate 2–4 cpm activity, suggesting they were measuring similar cyclic activity to that observed in this study. Welgan et al. also showed that stress and anger increase 2–4 cpm activity in the sigmoid colon (Welgan et al., 1985, 1988). Similarly, Rao et al. used low‐resolution manometry to show that experimentally induced psychological and physical stress both increase distal colonic motor activity, with longer lasting effects resulting from psychological stress (Rao et al., 1998).

The physiological role of this increase in distal colonic CMP activity in response to acute stress remains unclear. We hypothesize that increased sympathetic activity may allow myogenic mechanisms to become dominant, thereby increasing the CMP. Increased retrograde CMP activity may retain feces in the colon (Heitmann et al., 2021), potentially increasing the time available for absorption of salt and water, thereby retaining extracellular fluid as part of the neurohormonal stress response. Conversely, there was a predominance of antegrade activity in this analysis of preoperative patients, compared with previous studies of distal colonic CMP, which have described a predominance of retrograde events (Dinning et al., 2014; Lin, Du, et al., 2017; Pervez et al., 2020). It is interesting to speculate that this may be a potential explanation of the defecation response to fear commonly observed in animals and in humans (Sarna, 1991). The relationship of acute and chronic stress on colonic motility, and its effect on FGIDs such as irritable bowel syndrome requires further targeted investigation.

The invasive modality of recording colonic motility in this study precluded the recruitment of a large population of patients. However, significant differences in distal colorectal motor patterns have previously been demonstrated using HR manometry in similarly sized cohorts (Jaung et al., 2021; Keane et al., 2021). Emerging noninvasive techniques such as electrocolonography may provide an opportunity to study distal colonic motility in larger cohorts in future (Erickson et al., 2020). This study was also limited by not directly measuring preoperative anxiety levels or autonomic tone, therefore the relationship between distal colonic motility and anxiety requires further targeted investigation. We did not attempt to extract pulse rate data from colonic manometry recordings, as these are not consistently or reliably recorded, and the acquisition unit sampling frequency of 10 Hz precludes any meaningful analysis of heart rate variability (HRV). Further studies are now needed to correlate changes in colonic motility with measurements of autonomic tone such as HRV or complexity (Amalan et al., 2019; Mulder et al., 2020), and clinical anxiety scores (Burton et al., 2019). Although the healthy controls included in this study were neither age‐ nor sex‐matched, and received full bowel preparation, these data were also included to provide a comparison against physiological motility under standardized conditions and following a 700 kcal meal. Finally, this study was not powered or designed to assess the efficacy of preoperative carbohydrate drinks for improving postoperative outcomes, which has been well‐reviewed by recent meta‐analyses (Amer et al., 2017).

In summary, CMP activity in the distal colon increased with proximity to surgery, and this was amplified by ingestion of preoperative carbohydrate drinks. Further research should apply HR techniques to examine the potential effects of anxiety on CMP activity in healthy controls and other pathological states.

## CONFLICT OF INTEREST

GOG is a shareholder of Alimetry and holds intellectual property in the field of noninvasive gastric mapping. NP holds intellectual property in the field of gastric electrophysiology and is a shareholder in FlexiMap Ltd. No commercial financial support was received for this study. The other authors have no conflict of interest to declare.

## AUTHOR CONTRIBUTIONS

Cameron Wells: Conceptualization, methodology, formal analysis, validation, investigation, data curation, writing–original draft, visualization, and funding acquisition. Sameer Bhat: Formal analysis, data curation, and writing–original draft. Nira Paskaranandavadivel: Methodology, software, visualization, and writing–review and editing. Anthony Lin: Methodology, data curation, and writing–review and editing. Ryash Vather: Investigation, data curation, writing–review and editing, and funding acquisition. Chris Varghese: Formal analysis and writing–review and editing. James Penfold: Investigation and data curation. David Rowbotham: Investigation and resources. Phil Dinning: Investigation, data curation, writing–review and editing, supervision, and funding acquisition. Ian Bissett: Supervision, writing–review and editing, and funding acquisition. Greg O’Grady: Supervision, writing–review and editing, and funding acquisition.

## Supporting information



Fig S1

Fig S2

Fig S3
