# Supplementary figures and images for: Potential causes of the preoperative increase in the rectosigmoid cyclic motor pattern: A high‐resolution manometry study
Source: Physiol Rep. 2021 Nov 27;9(22):e15091. doi: 10.14814/phy2.15091 (PMC8627120; doi:10.14814/phy2.15091)

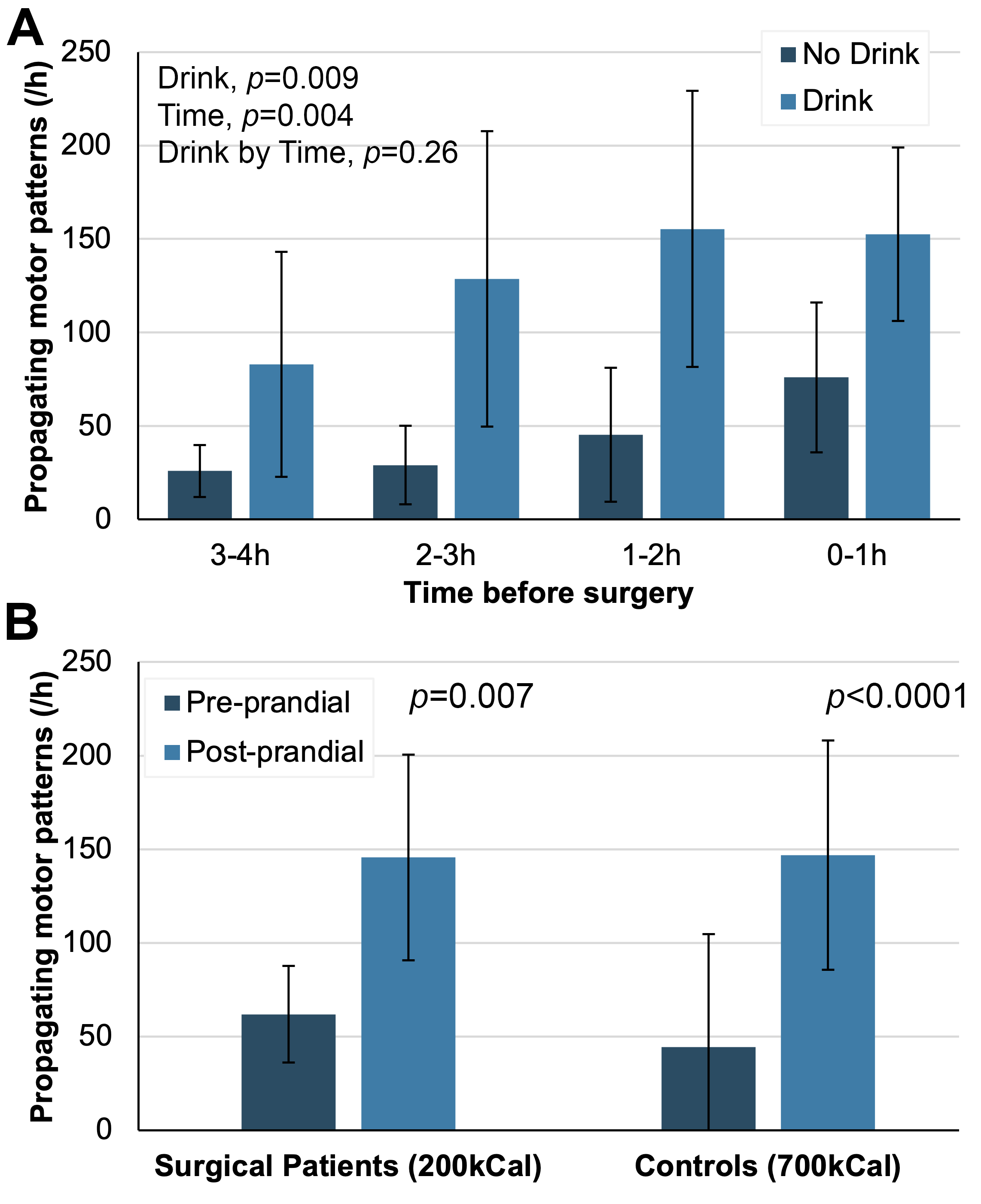

Supplement: Supplementary file 1 — Fig S1 [file PHY2-9-e15091-s001.tif]

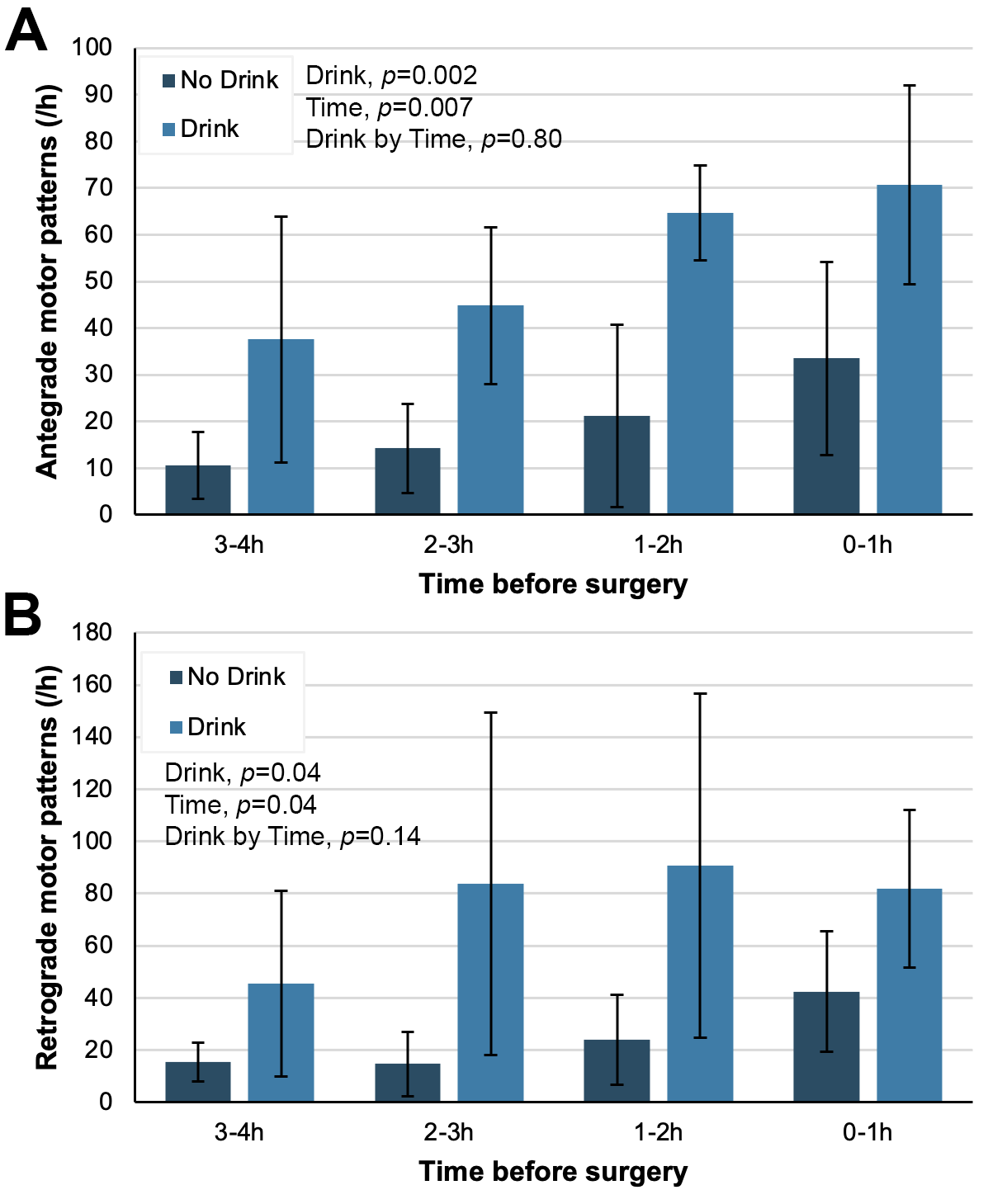

Supplement: Supplementary file 2 — Fig S2 [file PHY2-9-e15091-s003.tif]

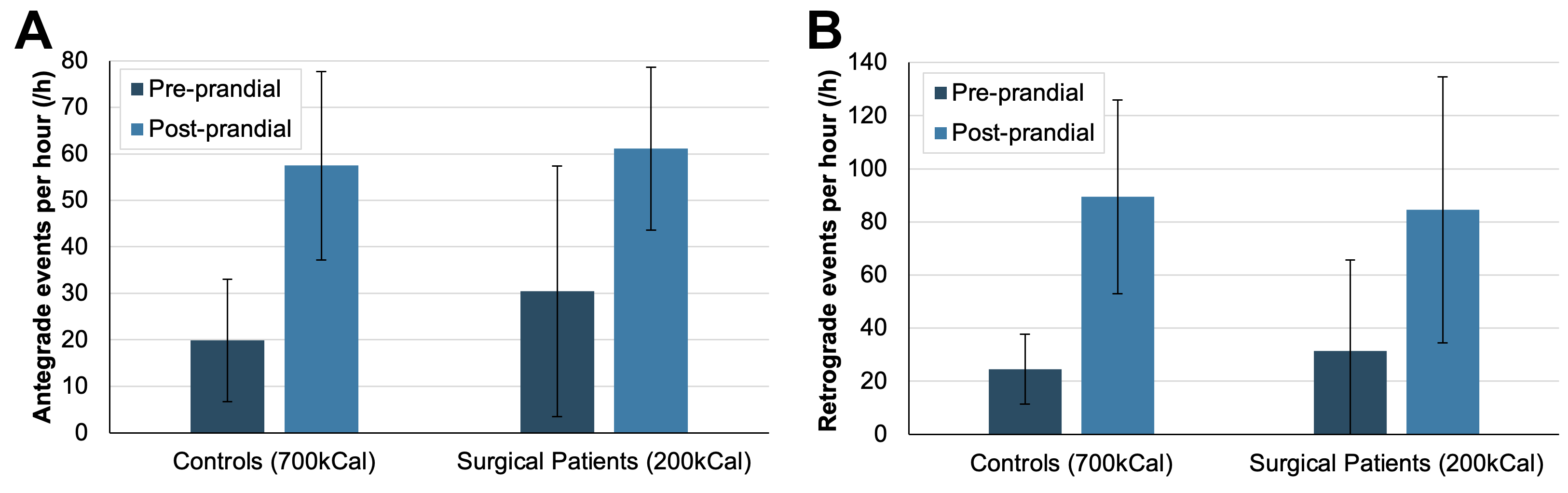

Supplement: Supplementary file 3 — Fig S3 [file PHY2-9-e15091-s002.tif]
